# Case Report: Adjuvant splenic irradiation therapy effectively eliminates donor-specific antibodies and reverses refractory early active antibody-mediated rejection after presensitized kidney transplantation

**DOI:** 10.3389/fimmu.2025.1670507

**Published:** 2025-09-04

**Authors:** Zhiliang Guo, Rula Sa, Guangyuan Zhao, Fuheng Che, Hui Guo, Lan Zhu, Gang Chen

**Affiliations:** Institute of Organ Transplantation, Tongji Hospital, Tongji Medical College, Huazhong University of Science and Technology, Key Laboratory of Organ Transplantation, Ministry of Education, Chinese Academy of Medical Sciences, NHC Key Laboratory of Organ Transplantation, Wuhan, China

**Keywords:** early active antibody-mediated rejection, donor-specific antibody, splenic irradiation, presensitized kidney transplantation, case report

## Abstract

In presensitized kidney transplantation with positive donor-specific antibody (DSA), the activation of immune memory responses leads to a significant increase in DSA levels, followed by early active antibody-mediated rejection (early aAMR). For some patients, it is difficult to eliminate DSA and reverse aAMR after conventional treatments such as plasmapheresis (PP) and intravenous immunoglobulin (IVIG). Here we report three cases of successful reversal of refractory early aAMR after DSA-positive presensitized kidney transplantation by using adjuvant splenic irradiation therapy. At 1 to 2 weeks after kidney transplantation, all three of our recipients experienced a significant increase in DSA levels, accompanied by deterioration of renal allograft function. The aAMR of two patients was diagnosed by renal biopsy, and the other was diagnosed clinically. After 5 to 11 sessions of PP/IVIG treatment, the DSA levels of all three patients failed to decrease, or even continued to rise. Therefore, in addition to PP/IVIG treatment, all three patients received 10 sessions of low-dose repetitive splenic irradiation (50cGy per session) as adjuvant therapy. As a result, the levels of all DSAs began a continuous decline, and renal function gradually returned to normal or approached normal. Eventually, all three patients recovered and were discharged from the hospital. During the 14- to 75-month follow-up period, the DSA of two patients became negative, while that of the remaining one patient remained at a low level. Renal function was stable during the follow-up period. Thus, when early aAMR that resists conventional treatment occurs after presensitized kidney transplantation, splenic irradiation may be an important adjuvant treatment option.

## Introduction

Early active antibody-mediated rejection (early aAMR) often occurs after presensitized kidney transplantation. This rejection may be mediated by preformed donor-specific antibodies (DSAs), or more likely caused by new DSA generated from the reactivation of memory B cells. According to published reports, the incidence rate of this early aAMR can be as high as 39% to 70% (1–3). To prevent the risk of early aAMR, plasmapheresis (PP) and intravenous immunoglobulin (IVIG) are often used before presensitized transplantation to reduce or clear preformed DSA, and rituximab (RTX) is used to deplete B cells to inhibit the rebound of DSA after transplantation (4–6). However, despite the use of these treatments, the risk of early aAMR after presensitized kidney transplantation remains relatively high. Once early aAMR occurs, most patients are routinely treated with PP/IVIG (7). However, for some patients, DSA is difficult to clear, or even reduce, after treatment, resulting in poor transplant outcome or even graft failure (8–10). How to deal with this early aAMR that resists conventional treatment is an urgent problem to be solved in the field of kidney transplantation.

The reasons for the resistance of early aAMR after presensitized kidney transplantation to conventional treatments such as PP/IVIG are principally that 1) the treatment cannot directly eliminate long-life plasma cells in the bone marrow, and these cells will continuously produce DSA (11); and 2) when the antigen is exposed again, the rapid response of memory B lymphocytes leads to the production of a large amount of DSA (12).

The spleen, as the largest immune organ in the body, is an important site for the activation of memory B cells and the production of antibodies. Animal experiments have demonstrated that the spleen is the main source of early post-transplant DSA in both sensitized and non-sensitized recipients (13). Splenectomy has been used in ABO-incompatible kidney transplantation and also in the treatment of AMR after kidney transplantation. However, in addition to the surgical risk associated with splenectomy itself, there is also a risk of systemic infection after the operation.

Splenic irradiation may have a similar effect to splenectomy. Orandi et al. first reported in 2016 the use of splenic irradiation to treat severe acute AMR in two presensitized recipients after kidney transplantation. After combined treatment with PP/IVIG, RTX, and eculizumab, good therapeutic effects were achieved. One patient had cleared DSA by 48 days after transplantation, while the DQ-DSA of the other patient decreased but persisted (14). As only two cases were reported and no subsequent reports on splenic irradiation for the treatment of acute AMR after kidney transplantation have been seen, there is currently a lack of sufficient evidence to evaluate the role of splenic irradiation in the treatment of early aAMR.

In the present study, we report three clinical cases of early aAMR after presensitized kidney transplantation. All recipients showed a significant increase in DSA combined with deterioration of renal function at 1–2 weeks after transplantation. The effects of multiple PP/IVIG treatments were poor, but after the addition of splenic irradiation to the treatment, DSA was significantly decreased, and the renal graft function recovered well.

## DSA monitoring

Single-antigen microbeads (LABScreen™ Single Antigen Beads, One Lambda Inc., Canoga Park, CA) were used to detect human leukocyte antigen (HLA) antibodies in our transplant center, and the dilution ratio of the serum used was 1:3 (15). The definition of a positive result was a detected mean fluorescence intensity (MFI) value >1,000.

## Case 1

The patient is a 54-year-old male with blood type O. In 2000, he underwent his first kidney transplant because of chronic glomerulonephritis and uremia. Ten years after transplantation, he resumed hemodialysis because of chronic failure of his renal allograft. In 2011, after the kidney graft was removed at the local hospital, all immunosuppressants were discontinued. In 2012, panel-reactive antibody (PRA) was found to be strongly positive (details unknown). In March 2018, the patient came to our hospital to register for a second kidney transplant, and the calculated PRA (cPRA) results showed that the sensitization to HLA-I and HLA-II was 97% and 92%, respectively. While waiting for kidney transplantation, oral immunosuppressant therapy was resumed: trough serum concentration of tacrolimus was maintained at 5–7 ng/ml, and mycophenolate mofetil (MMF) was given at a dose of 0.5g, q12 hr.

**Figure 1:**
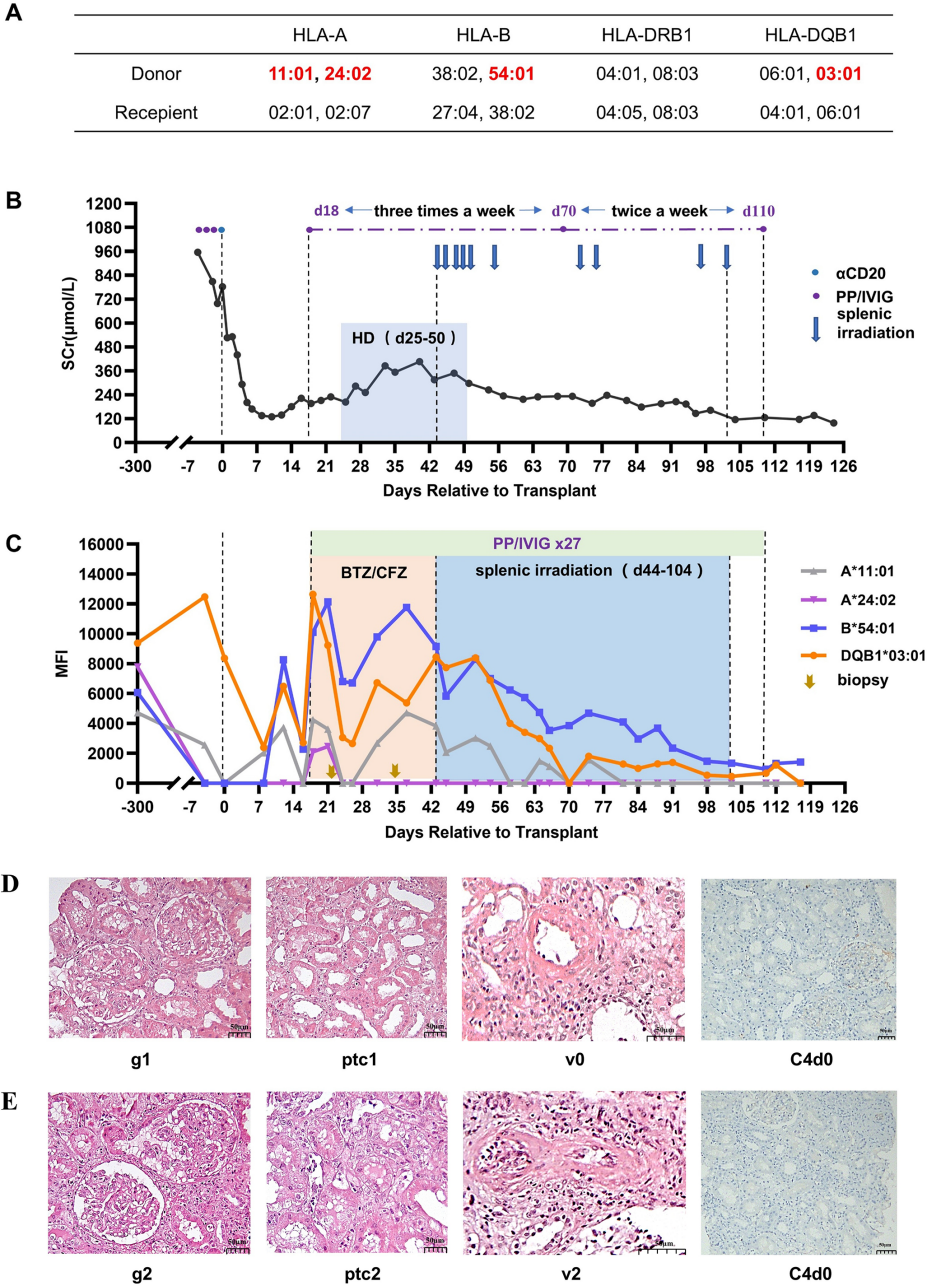
Treatment process and pathological results for Case 1. **(A)** HLA genotype results for the recipient and donor. **(B)** Changes in serum creatinine. **(C)** Changes in MFI values of DSAs. **(D)** Histopathological findings on day 22 and their Banff 2017 scores. **(E)** Histopathological findings on day 32. Hemodialysis (HD), bortezomib (BTZ), carfilzomib (CFZ), glomerulitis (g), peritubular capillaritis (ptc), intimal arteritis (v).

In January 2019, a potential donor of the same blood type emerged in our hospital. He was a 42-year-old male with brain trauma. The patient’s mismatch was four loci of HLA-A, B, DR, and DQ with him (**Figure 1A**). The latest antibody test results of the patient showed that two preformed DSAs existed, namely A11-DSA (MFI: 2,570) and DQ7-DSA (MFI: 12,471). In addition, the remaining two mismatch loci (A24 and B54) also had historical DSA. Since highly sensitized patients have few opportunities for transplantation, performing a DSA-positive deceased donor kidney transplant was considered for this patient.

To reduce the immunological risk, desensitization therapy was immediately administered to the patient: PP combined with IVIG. PP consisted of 1200 ml of fresh plasma plus 1500 ml of 5% albumin, and 20 g of IVIG was given each time. After three sessions of PP/IVIG treatment, the A11-DSA became negative, and the MFI value for DQ7-DSA dropped to 8,361. Complement-dependent cytotoxicity (CDC) was negative by flow cytometry. After receiving the kidney allocation, the patient underwent a second kidney transplant operation.

The immunosuppressive regimen used here is described in our previously published paper on kidney transplantation in presensitized patients (6). In brief, 200 mg of RTX was given before the transplantation, thymoglobulin (total dose 3 mg/kg) was administered as induction therapy, and IVIG was used continuously for 2 weeks after transplantation (20g/d for the first week, 10g/d for the second week). Methylprednisolone was given intravenously (500 mg/day, days 0-2), followed by oral doses of prednisone at 50 mg/day, which were then tapered every other day to a maintenance dose of 10 mg/day. Tacrolimus was started on day 2, with a targeted trough level of 7–10 ng/ml. MMF was administered at a dose of 1.5 g/day.

Renal function recovered immediately after transplantation, and the serum creatinine dropped to 129 μmol/L on day 11. HLA antibody monitoring on day 8 showed that DSA remained at a low level (the MFI of anti-DQ7 DSA decreased to 2,394, anti-A11 DSA increased slightly to 2,069). On day 13, the serum creatinine level increased to 180 μmol/L, and the DSA against A11, B54, and DQ7 showed some rebound. On day 17, the serum creatinine level further increased to 222 μmol/L. Meanwhile, the DSA against A24, A11, B54, and DQ7 all increased further, with the MFI values for B54-DSA and DQ7-DSA being >10,000. Considering the high possibility of the occurrence of aAMR, PP/IVIG treatment was initiated on day 18 (three times a week). A biopsy of the renal graft was performed on day 22. The pathology revealed moderate microvascular inflammation (MVI=2), and C4d was negative (**Figure 1D**). The diagnosis was mild aAMR. Bortezomib was added for 3 days (2.4 mg/d) starting on day 23. Despite the treatment, hemodialysis was started on day 25 because of oliguria and a significant increase in body weight.

On day 35, the MFI of the DSAs was still not reduced, and the renal function still had not recovered. Therefore, renal graft biopsy was again performed. The results indicated that the microvasculitis was more severe (MVI=4), and there was intimal arteritis accompanied by a slight cellulosic necrosis (Banff 2017 Schema, i0, t0, g2, ptc2, v2, C4d-, **Figure 1E**). Given the continuous aggravation of aAMR, carfilzomib (30 mg/d, for 2 days) was added to the PP/IVIG treatment, starting on day 36, but the results were still not satisfactory. After obtaining the patient’s informed consent, low-dose splenic irradiation was added to the PP/IVIG, starting on day 44, for a total of 10 sessions (50cGy per session). The first six sessions were intensive treatment (completed within 2 weeks), and the last four were supplementary treatment (**Figure 1C**). After splenic irradiation adjuvant therapy, the patient’s DSA steadily decreased, renal function gradually recovered, and hemodialysis was stopped on day 50 (**Figure 1B**). During the period of splenic irradiation treatment, the patient’s white blood cell count showed a general downward trend, while the number of lymphocytes did not significantly decrease. The patient experienced fatigue after multiple splenic irradiations, but no other obvious adverse reactions occurred.

The patient was cured and discharged on day 123. At discharge, the serum creatinine was 98 μmol/L, estimated glomerular filtration rate (eGFR) was 74.5mL/min/1.73m^2^, A11-DSA and A24-DSA were negative, and the MFI values of B54-DSA and DQ7-DSA had both dropped to <2,000 (**Figure 1C**). The patient has been followed up for 75 months thus far. Serum creatinine has remained stable at 90-110 μmol/L, urine protein has been maintained at 0.2-0.5 g/24h, and the DSAs became negative in the second year after transplantation and have remained so.

## Case 2

**Figure 2:**
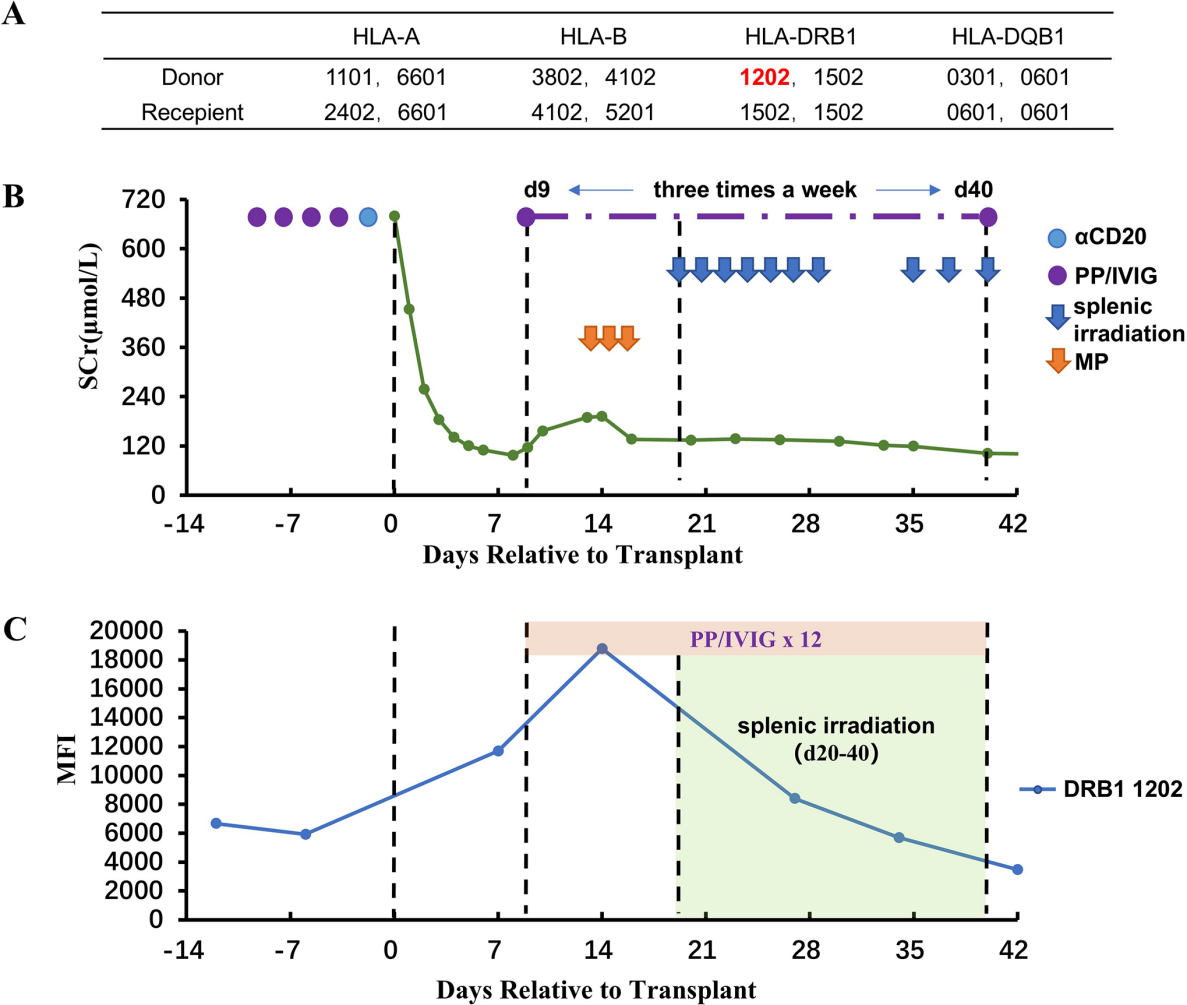
Renal function, DSA, and treatment after kidney transplantation for Case 2. **(A)** HLA genotype results for the patient and donor. **(B)** Changes in serum creatinine. **(C)** Changes in MFI values of DSA. Methylprednisolone (MP).

The patient is a 29-year-old female with blood type B. She has a history of blood transfusion and three pregnancies, and no previous history of transplantation. In February 2022, the patient came to our hospital for a living-related kidney transplantation because of uremia (unknown primary disease). The cPRA values were 12.7% for HLA-I and 68.3% for HLA-II. The donor was the patient’s mother, age 58 years, with the same blood type and HLA haploid matching (**Figure 2A**). Preformed DSA was identified as targeting DR12, and the MFI was 6,669.

Before transplantation, the patient was given a basic desensitization regimen of PP+IVIG+RTX and was orally administered tacrolimus + MMF at the same time. After four sessions of PP/IVIG treatment, the MFI value of the DR12-DSA have decreased slightly (5,923), and the Flow-CDC test result was negative. The kidney transplantation was then performed. The perioperative immunosuppressive regimen was almost the same as that of Case 1.

After transplantation, the serum creatinine level dropped rapidly to 97 μmol/L on day 8. However, a rebound in serum creatinine began on day 9, and at the same time, the MFI value for DR12-DSA was significantly elevated (11,692). Color Doppler ultrasound of the renal allograft indicated an enlargement in size (13.5 X 5.9 cm, vs. 11.4 X 5.0 cm on day 1). PP/IVIG treatment was initiated immediately (three times a week), and methylprednisolone (500/500/300 mg) was given for 3 consecutive days beginning on day 13. However, DR12-DSA showed a continued increase on day 14 (MFI:18,778). Moreover, the serum creatinine level had also been continuously rising (from 97 μmol/L to 192 μmol/L). Therefore, on day 20 (after five sessions of PP/IVIG treatment), splenic irradiation therapy was added, for a total of 10 sessions (50 cGy per session). The protocol was similar to that for Case 1 (**Figure 2B**). After splenic irradiation was added, the patient’s DSA and serum creatinine decreased steadily without rebound. During the period of splenic irradiation treatment, the patient’s white blood cell and lymphocyte counts did not show any significant decrease. After multiple splenic irradiations, the patient experienced fatigue and a slight impairment of appetite. No other obvious adverse reactions occurred.

The patient was discharged on day 43. At discharge, the serum creatinine was 102 μmol/L, eGFR was 64.2mL/min/1.73m^2^, and the MFI value of DR12-DSA was 3,475 (**Figure 2C**). This patient did not undergo renal biopsy because of the risk of menstrual bleeding after transplantation. During the follow-up period of 40 months, the serum creatinine level has fluctuated between 110 and 130 μmol/L, and there has been no proteinuria. In addition, the DR12-DSA became negative in the 4th month after transplantation. No serious infections or other adverse effects occurred during the follow-up period.

## Case 3

The patient is a 37-year-old female with blood type B. In 2013, her first kidney transplant was performed at another hospital in response to uremia caused by membranoproliferative glomerulonephritis. In 2015, because of the recurrence of the primary disease, the renal graft lost function, and hemodialysis was resumed. The graft was removed at the transplant hospital, and then all immunosuppressants were discontinued. Eight years later (in 2023), the patient registered for a second kidney transplantation in our hospital. The cPRA result showed high sensitization: 94% for HLA-I and 91% for HLA-II. While the patient was waiting for kidney transplantation, oral immunosuppressants were resumed: The tacrolimus trough level was maintained at 5–7 ng/ml, and MMF was given at a dose of 0.5 g, q12 hr.

**Figure 3:**
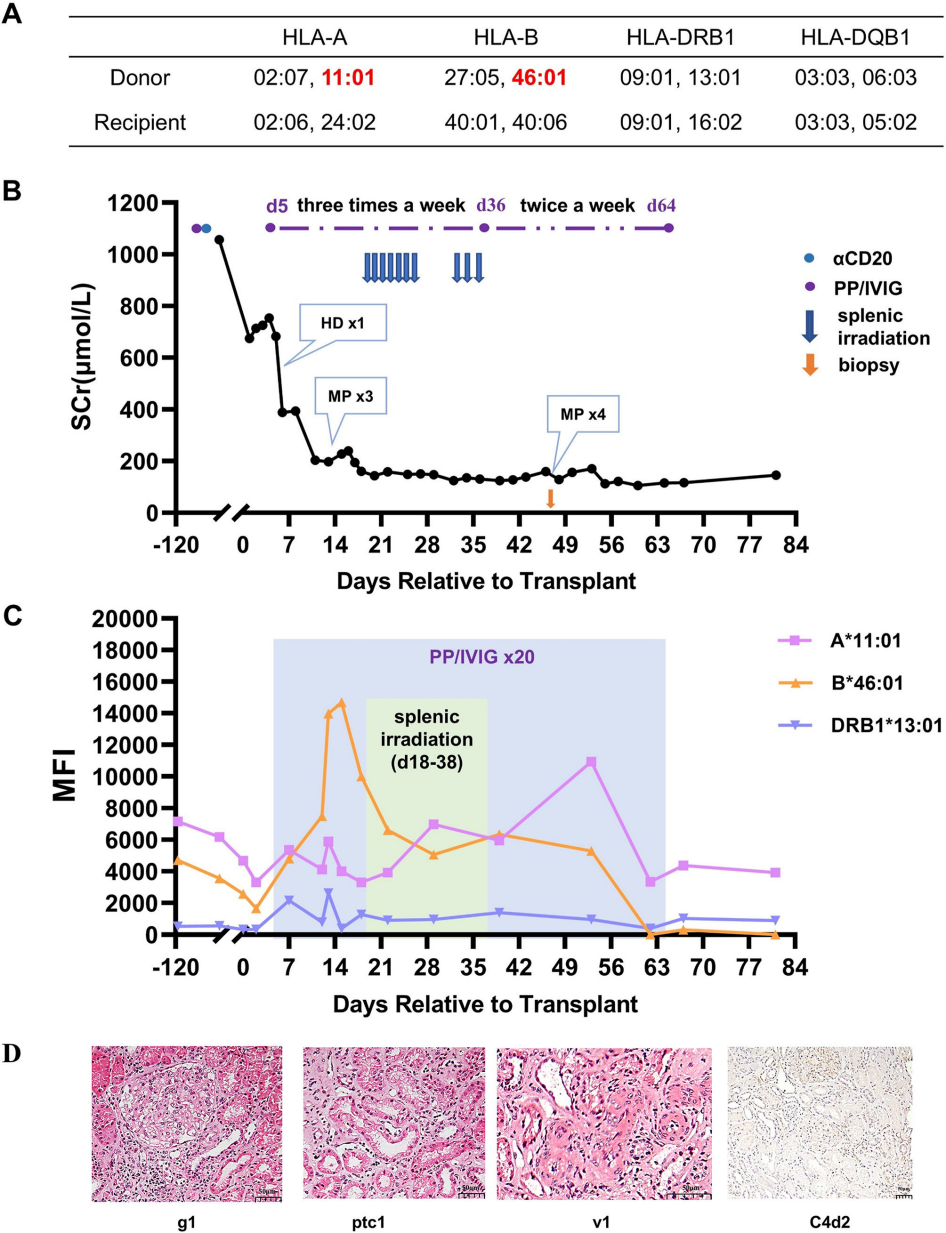
Treatment process and pathological results for Case 3. **(A)** HLA genotype results for the patient and donor. **(B)** Changes in serum creatinine. **(C)** Changes in MFI values of DSAs. **(D)** Histopathological features of the renal allograft on day 48 and their Banff 2019 scores. Hemodialysis (HD), methylprednisolone (MP), glomerulitis (g), peritubular capillaritis (ptc), intimal arteritis (v).

In February 2024, the patient received a kidney through the China Organ Transplant Response System. The donor was a 46-year-old male, and the cause of death was cerebral hemorrhage. There were five mismatched loci for HLA-A, B, DR, and DQ (**Figure 3A**). The latest antibody test results showed that two preformed DSAs existed, namely A11-DSA (MFI: 6,174) and B46-DSA (MFI: 3,562), and the Flow-CDC result was negative. Before transplantation, an emergency PP/IVIG treatment was given to the patient. DSA retest results showed that the MFI value of A11-DSA dropped to 4,680 and that of the B46-DSA decreased to 2,578.

The overall perioperative immunosuppression protocol for Case 3 was almost the same as that for Case 1. The postoperative urine output was normal, but the recovery of the renal allograft function was slow. On day 5, the serum creatinine was still 682 μmol/L, and hemodialysis was performed once. On day 7, the preformed DSAs began to rebound slightly, and DR13-DSA became positive. PP/IVIG treatment was then initiated immediately (three times a week). On day 13, the patient’s serum creatinine was maintained at approximately 200 μmol/L, but the urine output decreased from that observed previously. Doppler ultrasound indicated that the blood flow resistance of the renal graft had increased. Early T-cell-mediated rejection was clinically diagnosed. Three doses of methylprednisolone (500/500/300 mg) pulse therapy were given. Subsequently, the creatinine decreased to 160 μmol/L. After 5 sessions of PP/IVIG treatment, the patient’s DSA remained at a relatively high level, especially the MFI of B46-DSA, which was >10,000.

To promote the clearance of DSA, with the patient’s consent, splenic irradiation therapy was added to PP/IVIG treatment. The basic regimen was similar to that of the previous two cases. After 10 sessions of splenic irradiation, the DSAs began to gradually decrease. Meanwhile, the renal function further improved, with serum creatinine dropping to 120-140 μmol/L. During the treatment with splenic irradiation, the patient’s white blood cell counts significantly decreased, while the lymphocyte count remained stable. The patient experienced a decrease in appetite, but no other obvious adverse reactions occurred.

To evaluate whether DSA caused injury to the allograft, a renal biopsy was performed on day 48. The results indicated acute mixed rejection (Banff 2019, i2, t1, g1, v1, ptc1, C4d2, **Figure 3D**). Therefore, PP/IVIG treatment was continued, and four additional doses of methylprednisolone (500/500/300/300 mg) were given. The patient was finally discharged on day 68, with a serum creatinine level of 116 μmol/L (**Figure 3B**) and eGFR was 51.9mL/min/1.73m^2^. At discharge, the MFI value for A11-DSA was 3,932, and both B46-DSA and DR13-DSA had become negative (**Figure 3C**).

The patient has been followed up for 14 months. The serum creatinine level has fluctuated between 130 and 150 μmol/L, and proteinuria has remained negative. In addition, the A11-DSA has remained at a low level (MFI:3,441), and no other DSA has occurred.

## Discussion

In presensitized kidney transplantation, preformed DSA is a dominant risk factor for AMR. At the same time, memory B cells in the recipient that can respond to the donor’s mismatched HLA loci are a latent risk factor that cannot be ignored. Reducing the intensity of DSAs through PP/IVIG treatment and requiring a negative flow-CDC test result before transplantation can effectively avoid hyperacute rejection and significant acute antibody-mediated injury. However, the secondary immune response that occurs when memory B cells are exposed to the same or similar alloantigen after transplantation can significantly increase the level of preformed DSA and even generate new DSAs targeting other potential sensitization sites, thereby representing an important cause of early aAMR after presensitized kidney transplantation.

In the present study, all three of our patients showed a significant increase in preformed DSA levels 1–2 weeks after transplantation. Furthermore, in Case 1, there was only one preformed DSA detected before transplantation; however, by ~2 weeks after transplantation, DSAs targeting the other three loci had been identified and began to increase significantly. These DSAs had occurred before the transplantation but turned negative on their own during the waiting period for the transplantation, perhaps thanks to a secondary immune response of memory B cells after transplantation. Therefore, during the process of presensitized kidney transplantation, it is not only necessary to pay attention to the sensitized HLA loci and DSA levels at the time of transplantation, but also to attach importance to the degree of historical HLA sensitization and the sensitized loci of the recipients.

There is currently no standard treatment regimen for early aAMR in presensitized kidney transplantation. Although PP/IVIG+RTX has achieved a certain success in the treatment of early aAMR after kidney transplantation (16–18), its therapeutic effect on some patients with refractory AMR is limited. In the present study, after 11 sessions of PP/IVIG and the additional treatment with proteasome inhibitors in Case 1, the DSA remained high, and the renal graft function continued to deteriorate. Both Case 2 and Case 3 patients were unable to effectively reduce the DSA level despite five sessions of PP/IVIG treatment. Therefore, for such patients with intractable early aAMR, new treatment methods need to be sought to assist in clearing DSA and preventing antibody rebound after clearance.

The spleen is the largest lymphoid organ in the body and is closely related to the B-cell immune response (19). Splenectomy was once used in the treatment of early aAMR after kidney transplantation and showed definite effects (20, 21). Among the 100 presensitized patients reported by Locke et al., 8 individuals developed severe aAMR after kidney transplantation; there was no significant improvement after treatment with PP/IVIG. Eventually, three transplant patients who did not undergo splenectomy lost their renal grafts. In contrast, the other five patients who underwent splenectomy exhibited significant therapeutic effects and no graft loss (20). However, in addition to the risks of splenectomy itself, there is also a risk of systemic infection after splenectomy. Kyaw et al. collected relevant information for 1,648 patients who underwent splenectomy. Among them, 350 patients (21.2%) were hospitalized because of severe infection after splenectomy. The highest incidence of infection was within 3 years after splenectomy, and the older the age, the higher the risk of infection (22).

Orandi et al. first reported two cases of early severe AMR after kidney transplantation when splenic irradiation was used instead of splenectomy. Both sensitized kidney transplant recipients developed severe AMR one week after the operation. On the basis of PP/IVIG, RTX, and eculizumab treatment, splenic irradiation therapy was initiated on days 8 and 9, respectively. After they received 11 and 4 sessions of splenic irradiation treatment, respectively, the AMR of both patients was significantly reversed (14). Since these two individuals began splenic irradiation treatment at the initial stage of AMR treatment and in combination with PP/IVIG, RTX, and eculizumab, the effect of splenic irradiation has not yet been clearly verified.

In the current study, all three patients received at least five sessions of PP/IVIG treatment before splenic irradiation therapy was begun. The DSA remained at a high level or even continued to increase, and the renal graft function did not improve, continuing to deteriorate. After splenic irradiation treatment was added, the DSA declined without rebound, and the renal graft function gradually recovered. These three cases clearly reinforce the effectiveness of splenic irradiation treatment. PP/IVIG can reduce DSA levels and have a certain inhibitory effect on the rebound of DSA. However, since it does not directly target plasma cells, its treatment effect is not satisfactory for early aAMR in some patients. Adding splenic irradiation can reduce or inhibit plasma cells and memory B cells, which can further weaken the ability of DSA production, thereby possibly achieving the goal of reducing or even eliminating DSA.

Glasow et al. have demonstrated through animal experiments that splenic irradiation can reduce the number of plasma cells in the circulation, and the specific therapeutic effect is related to the radiation dose and the course of treatment (23). An animal study using a murine heart transplant model has demonstrated that the spleen rather than bone marrow is the major source of donor-reactive alloantibody early after transplantation in both sensitized and non-sensitized recipients (13). The splenic irradiation is widely used in the treatment of chronic leukemia and myeloproliferative disorders. The mechanism of its action has been reported to be related to direct cell killing, immune modulation via changes in lymphocyte subsets or cytokine induction (24). The mechanism by which splenic irradiation is used for desensitization therapy in the field of kidney transplantation remains unclear. The effect of splenic irradiation is not necessarily equivalent to splenectomy. The spleen is not only an important site for the activation of memory B cells but also an important transfer station for the lymphocyte circulation. Although splenectomy directly reduces the intensity of a B-cell response to a large extent, it cannot prevent the activation of memory B cells in other place of the body, such as the lymph nodes (25). In contrast, repeated low-dose splenic irradiation not only directly prevents the activation of B cells in the spleen but may also continuously interfere with the lymphocytes circulating to the spleen during treatment. Therefore, it may offer certain advantages over splenectomy.

We have previously attempted to use splenic irradiation as an adjuvant therapy for chronic aAMR after kidney transplantation. The results showed that adding splenic irradiation to the PP/IVIG treatment could reduce the level of DSAs in the circulation to a certain extent, alleviate glomerulitis and peritubular capillaritis, and help slow down the progression of chronic aAMR. However, the decreased DSAs all showed a significant rebound within one year (15). In the present study, three cases adopted a similar treatment protocol, but the DSAs did not show a significant rebound after the treatment was stopped. This suggests that compared with the treatment of chronic aAMR, splenic irradiation may have a better therapeutic effect on early aAMR after kidney transplantation. We speculate that the reasons for the different therapeutic effects are as follows: chronic aAMR is caused by the long-term action of *de novo* DSA (dnDSA). The plasma cells that produce dnDSA are mainly long-lived plasma cells that reside in the bone marrow, while the plasma cells in the spleen or circulation play a relatively limited role in the process; when early aAMR occurs in presensitized kidney transplantation, it is mainly due to the memory B lymphocytes in the spleen or lymph nodes being stimulated by the corresponding allogeneic antigens and differentiating into plasma cells, thereby generating a large amount of induced DSA. Therefore, the treatment effect of splenic irradiation may be better. These speculations still require further studies to be confirmed.

At present, the reports on the treatment of early aAMR after kidney transplantation with splenic irradiation are very limited. In addition, because of the particularity of early aAMR, it is difficult to conduct prospective randomized controlled studies. The exact effect and mechanism of splenic irradiation are therefore still difficult to determine. However, in this study we were able to achieve satisfactory results in all three cases when splenic irradiation was used, suggesting that it may serve as an important adjuvant treatment option in the future when early aAMR resistant to conventional treatment occurs after presensitized kidney transplantation.

## Data Availability

The original contributions presented in the study are included in the article/supplementary files, further inquiries can be directed to the corresponding author/s.
